# Contact Resistance and Channel Conductance of Graphene Field-Effect Transistors under Low-Energy Electron Irradiation

**DOI:** 10.3390/nano6110206

**Published:** 2016-11-10

**Authors:** Filippo Giubileo, Antonio Di Bartolomeo, Nadia Martucciello, Francesco Romeo, Laura Iemmo, Paola Romano, Maurizio Passacantando

**Affiliations:** 1 CNR-SPIN Salerno, via Giovanni Paolo II, 132, 84084 Fisciano, Italy; nadia.martucciello@spin.cnr.it; 2 Dipartimento di Fisica, Università di Salerno, via Giovanni Paolo II, 132, 84084 Fisciano, Italy; dibant@sa.infn.it (A.D.B.); fromeo@sa.infn.it (F.R.); liemmo@unisa.it (L.I.); 3 Dipartimento di Scienze e Tecnologie, Università del Sannio, via Port’Arsa 11, 82100 Benevento, Italy; promano@unisannio.it; 4 Dipartimento di Scienze Fisiche e Chimiche, Università dell’Aquila, Via Vetoio, 67100 L’Aquila, Italy; maurizio.passacantando@aquila.infn.it

**Keywords:** graphene, field-effect transistor, electron irradiation, contact resistance

## Abstract

We studied the effects of low-energy electron beam irradiation up to 10 keV on graphene-based field effect transistors. We fabricated metallic bilayer electrodes to contact mono- and bi-layer graphene flakes on SiO_2_, obtaining specific contact resistivity $\rho_{c}\approx 19\;k\Omega \cdot {µm}^{2}$ and carrier mobility as high as 4000 cm^2^·V^−1^·s^−1^. By using a highly doped p-Si/SiO_2_ substrate as the back gate, we analyzed the transport properties of the device and the dependence on the pressure and on the electron bombardment. We demonstrate herein that low energy irradiation is detrimental to the transistor current capability, resulting in an increase in contact resistance and a reduction in carrier mobility, even at electron doses as low as 30 e^−^/nm^2^. We also show that irradiated devices recover their pristine state after few repeated electrical measurements.

## 1. Introduction

Graphene is a promising candidate for future nanoelectronics and has been attracting an enormous amount of attention from the scientific community since 2004, when graphene flakes were exfoliated from graphite for the first time in Manchester [1]. Due to physical limits of Si-technology downscaling, the carbon-based electronics is considered a possible option [2] towards the post-silicon era. Carbon nanotubes (CNTs) have been largely studied in the last two decades but two principal drawbacks have limited their applicability: uncontrollable chirality causing both metallic and semiconducting nanotubes in fabrication processes and the difficulty of correctly placing a large number of nanotubes in integrated circuits. Graphene has reignited the idea of carbon-based electronics, offering unmatched properties such as a linear dispersion relation, with electrons behaving as massless Dirac fermions [3], a very high carrier mobility [4], and a superior current density capability [5]. Graphene is already a reality in applications such as gas sensors [6], photodetectors [7], solar cells [8], heterojunctions [9], and field-effect transistors [10,11].

From an experimental viewpoint, the use of scanning electron microscopy (SEM), transmission electron microscopy (TEM), electron beam lithography (EBL), and focus ion beam (FIB) processing in an ultra-high vacuum represents a necessary step for the fabrication and characterization of graphene-based devices. Consequently, graphene devices during fabrication or under test are necessarily exposed to a high vacuum and electron irradiation, which may considerably affect their electronic properties.

Several experiments have shown that the irradiation of energetic particles, such as electrons [12,13,14,15,16] and ions [17,18], can induce defects and damages in graphene and cause a severe modifications of its properties.

Raman spectroscopy has been largely used to study electron-beam induced structural modifications [19,20,21], or formation of nanocrystalline and amorphous carbon [18,22], and to correlate the reduction in 1/f noise in graphene devices with an increasing concentration of defects [23]. The shape and relative magnitude of a D peak, as well as the shift of the G peak, have been used to quantitatively evaluate the damage and the strain induced by a very low energy e-beam [24]. Raman and Auger electron spectroscopy have shown that e-beam irradiation can selectively remove graphene layers and induce chemical reactions and structural transformations [20,21]. The interaction of an e-beam with water adsorbates on the graphene surface has been also proposed for the hydrogenation of graphene [25,26]. However, Raman spectroscopy is unable to reveal all the effects of e-beam irradiation, and electrical measurements are needed to check for possible modifications of transport properties. Despite that, electronic transport properties of irradiated graphene devices have not yet been deeply investigated [27,28]. The negative shift of the Dirac point has been reported as an effect of e-beam-induced n-doping. The comparison with the case of suspended graphene has also evidenced the importance of the substrate [27]: It has been demonstrated in particular that e-beam irradiation of graphene field effect transistors (GFETs) modifies the substrate band bending and results in localized n-doping of graphene, which creates graphene p–n junctions working as a photovoltaic device [29].

In this paper, we study the modification of electronic transport properties of GFETs upon exposure to electron beam irradiation for scanning electron microscopy imaging with an acceleration energy up to 10 keV. An optimized fabrication process has been developed to obtain devices characterized by specific contact resistivity $\rho_{c}\approx 19\;k\Omega \cdot µm$^2^ and a carrier mobility as high as 4000 cm^2^·V^−1^·s^−1^ on a Si/SiO_2_ substrate. Electron irradiation affects the transistor current drive capability by reducing the carrier mobility and increasing the channel and contact resistance. We also show that, for low energy electron irradiation, the conditions of pristine devices are almost restored by successive gate voltage sweeps.

## 2. Materials and Methods

Graphene flakes were obtained from highly oriented pyrolytic graphite (from NGS Naturgraphit GmbH) by a scotch tape method and were placed on standard p-Si/SiO_2_ (300-nm-thick) substrates. After optical identification, the mono- or bi-layer nature of the flakes were confirmed by Raman spectroscopy. Metal contacts to selected graphene flakes were realized by means of electron beam lithography and magnetron sputtering techniques. Spin coating of approximately 400 nm PMMA-A7 (poly-methyl methacrylate) at 4000 rpm was performed on the sample, and it was successively exposed by an EBL system, Raith Elphy Plus (Dortmund, Germany). Methyl isobutyl ketone and then isopropanol was used as a developer. The metal electrodes were fabricated by a three cathode RF Sputtering Magnetron (by MRC Inc., Orangeburg, NY, USA) for in-situ multilayer deposition working at 10^−7^ mbar base pressure. The graphene flakes were contacted by a Nb/Au metallic bilayer (15 nm Nb/25 nm Au) with niobium contacting the graphene and gold working as a cap layer to prevent Nb oxidation and favor electrical connection with the probe tips. Metallic leads were sputtered at low power density (<0.7 W·cm^−2^) and small deposition rates (0.3 nm/s for Nb and 1.2 nm/s for Au) to prevent graphene damages.

Electrical characterization was performed by means of a Janis Research ST-500 cryogenic probe station (Woburn, MA, USA) connected to a Keithley 4200 (Beaverton, OR, USA) Semiconductor Characterization System (SCS) working in wide ranges of current (100 fA to 0.1 A) and voltage (10 μV to 200 V). To study the effect of e-beam irradiation on transistors, the SCS was connected to a scanning electron microscope Zeiss LEO 1430 (Oberkochen, Germany) equipped with Kleindeik nanomanipulators MM3A (Reutlingen, Germany), which allowed in-situ electrical measurements with the sample inside the high-vacuum SEM chamber to prevent adsorbate contamination.

## 3. Results and Discussion

### 3.1. Contact Resistance

**Figure 1:**
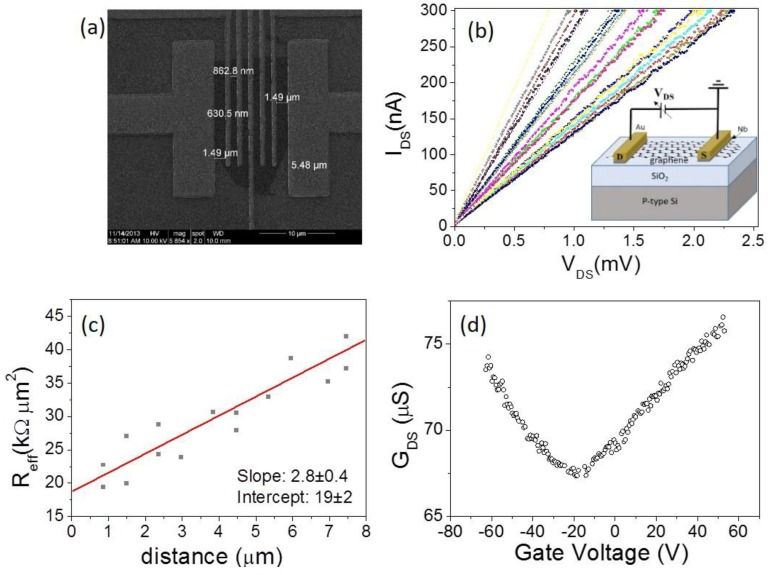
(**a**) A transfer length method (TLM) device with Nb(15 nm)/Au(25 nm) contacts. (**b**) Current–voltage characteristics measured for all possible two-lead combinations in the TLM device, at *V*_Gate_ = 0 V; inset: scheme of the device. (**c**) TLM plot of *R*_eff_ (L) at *V*_Gate_ = 0 V. (**d**) Transfer characteristic of one of the back-gated transistors of (a) in the range −60 V < *V*_Gate_ < +60 V.

In order to characterize the contact resistance, we designed a device with standard geometry to apply the transfer length method (TLM), a structure consisting of a series of spaced electrodes up to 10 µm apart (Figure 1a).

The two-probe current–voltage characteristic of the channel (*I*_DS_ vs. *V*_DS_) has been measured for each possible combination of contacts: the drain current (*I*_DS_) linearly increases with source-drain voltage (*V*_DS_), which is a typical behavior at low bias (Figure 1b). According to the TLM, we can extract the specific contact resistivity $\rho_{c}$ by evaluating (for the general situation of irregular shaped flakes) the intercept of a plot of *R*_eff_ vs. *L* [30], with *L* the separation between the two electrodes, and

$$R_{\mathrm{eff}}=R{\left(\frac{1}{W_{1}d_{1}}+\frac{1}{W_{2}d_{2}}\right)}^{-1}$$

where W*_i_* and d*_i_* (for *i* = 1, 2 ) indicate width and length of each contact, respectively. From the linear fitting of *R*_eff_ vs. *L* (see Figure 1c), we find $\rho_{c}=19\pm 2\;k\Omega \cdot {\mu m}^{2}$, an intermediate value compared with previously reported values of 7 kΩ·μm^2^ for Ni and 30 kΩ·μm^2^ for Ti [31].

**Figure 2:**
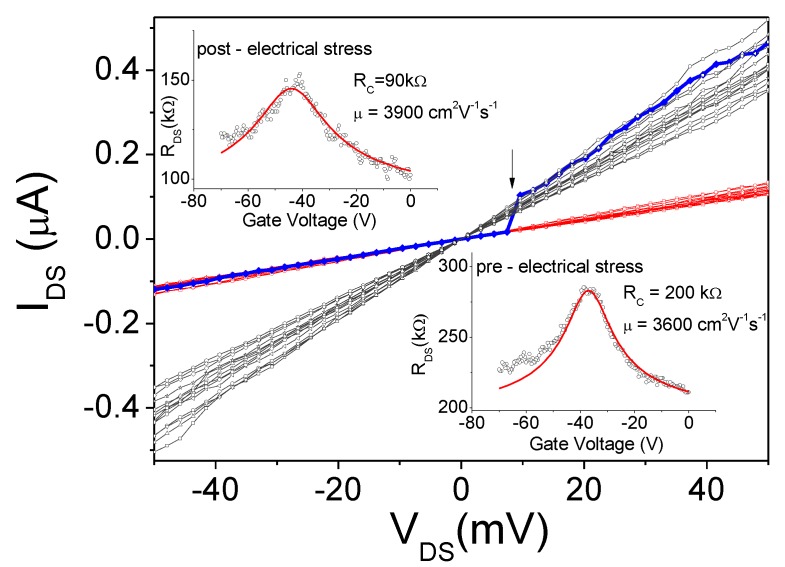
Output characteristics (*I*_DS_ vs. *V*_DS_) and transfer characteristics (*R*_DS_ vs. *V*_Gate_ in the insets) measured before and after the stabilization of the device due to electrical stress. Black arrow indicates the switch from higher to lower total resistance. Continuous (red) lines in the insets represent the numerical simulations obtained from the model of [32]. The contact resistance *R*_contact_ is abbreviated as *R*_C_ in the figures.

We also tested the current modulation of this device when used as a field effect transistor with the Si substrate as the back-gate electrode. In Figure 1d, we report the transfer characteristic *G*_DS_ vs. *V*_Gate_ in which the channel conductance *G*_DS_ is measured as a function of the gate voltage *V*_Gate_ between a couple of electrodes biased at *V*_DS_ = 0.5 mV. The conductance clearly shows a minimum at *V*_Gate_ = −15 V, corresponding to the charge neutrality point (Dirac point). The negative value indicates that the graphene is *n*-doped. The device was measured as produced, without any electrical annealing (stress), which is suitable for inducing the desorption of surface contaminants and improving the metal-graphene coupling, thus reducing the contact resistance [5]. In Figure 2, we show the output characteristics (*I*_DS_ vs. *V*_DS_ for several *V*_Gate_ values in the range −60 V to +60 V) and the transfer characteristic (at fixed *V*_DS_) measured before and after an electrical stress event that stabilize the device improving its performances. The black arrow in the figure identifies the voltage at which the device was suddenly modified, switching from a total resistance of about 250 kΩ to 150 kΩ, for the effect of current annealing. After such modification, the device was routinely measured, showing extreme stability without further modification of the total resistance *R*_DS_, which we report as a function of *V*_Gate_ in the insets of Figure 2. *R*_DS_ is the series of the contact resistance and the channel resistance, *R*_DS_ = *R*_C_ + *R*_channel_, where the channel resistance can be expressed as $R_{\mathrm{channel}}=\frac{L/W}{\mu \;n(V_{\mathrm{bg}})\;q}$ with *L* and *W* the length and width of the channel, respectively, μ is the carrier mobility, and *q* is the unit charge [32]. The total carrier concentration can be written $\left(V_{\mathrm{bg}}^{*}\right)=\sqrt{n_{\mathrm{ind}}^{2}+n_{0}^{2}}$, where $V_{\mathrm{bg}}^{*}$ is the back-gate voltage with respect to the Dirac voltage ($V_{\mathrm{bg}}^{*}=V_{\mathrm{bg}}-V_{\mathrm{Dirac}}$), *n*_0_ is the intrinsic carrier concentration, and *n*_ind_ is the carrier concentration induced by the back gate. *n*_ind_ can be expressed in terms of gate oxide capacitance as $n_{\mathrm{ind}}\left(V_{\mathrm{bg}}^{*}\right)=C_{\mathrm{ox}}V_{\mathrm{bg}}^{*}/q$. This model, adapted to the experimental data *R* vs. *V*_Gate_ allows for the extraction of the contact resistance and carrier mobility as fitting parameters.

Using the transfer characteristics measured before and after the electrical stress, we found that the contact resistance improved (reduced from 200 kΩ to 90 kΩ), while the carrier mobility increased from 3600 V^2^·cm^−1^·s^−1^ to 3900 V^2^·cm^−1^·s^−1^. The electrical stress increased the graphene–metal coupling and worked to clean the channel. The mobility values are comparable to values already reported for Nb-contacted GFETs [33]. We also noticed that the characteristic measured before the electrical stress showed an asymmetric shape, with the *p*-branch clearly away from the expected theoretical behavior. This can be explained in terms of the reduced coupling between the Nb electrode and the graphene channel (corresponding to large contact resistance), a situation that can cause asymmetry, a double dip, or both in such curves as reported in [33,34]. The improvement of the contact after electrical stress, resulting in better coupling between Nb and graphene, removed the asymmetry. Comparing the channel resistances that were extracted as *R*_DS_ − *R*_contact_ we also confirmed the improvement of the channel resistance.

**Figure 3:**
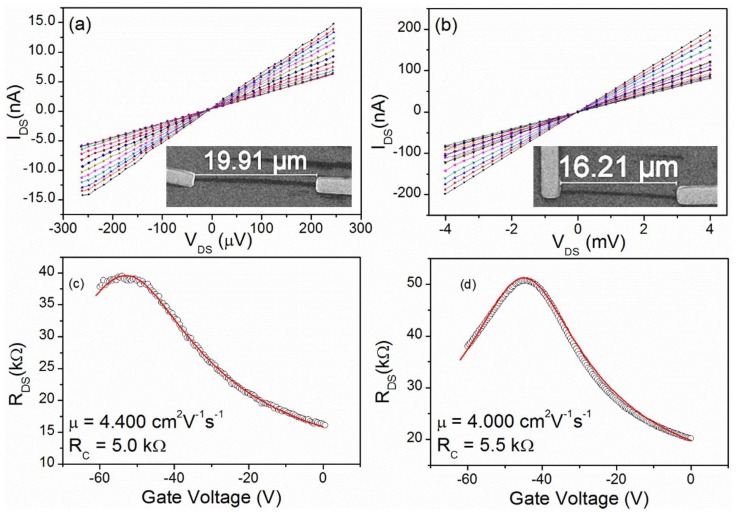
Electrical characterization under a high vacuum of two devices produced on the same substrate. (**a**,**b**) *I*_DS_ vs. *V*_DS_ curves for the devices shown in the insets with dimensions 19.9 µm × 0.7 µm and 16.2 µm × 0.3 µm, respectively. Curves are measured for different gate voltages in the range −60 V < *V*_Gate_ < 0 V with steps of 5 V. (**c**,**d**) *R*_DS_ vs. *V*_Gate_ curves measured at *V*_DS_ = 1 mV for the devices of Figure 3a,b, respectively. The solid (red) lines are the fitted model of [32] with the parameters listed in the plots.

In Figure 3, we show the electrical characterization of two other representative devices of the dozen produced in the same batch, after stabilization by electrical stress. The curves of Figure 3a,b are the output characteristics measured in a high vacuum (10^−7^ mbar) for different gate voltage values. The ohmic nature of the contacts is confirmed by the linearity of such characteristics.

In Figure 3c,d, we show the corresponding transfer characteristics measured at fixed drain-source bias *V*_DS_ = 1 mV. Remarkably, the current annealing and the long high vacuum storage produced very stable devices with low contact resistance (5.0 kΩ < *R*_contact_ < 5.5 kΩ). The high fabrication quality is confirmed by the small contact resistance, the low noise, and the high carrier mobility, which is 4000 V^2^·cm^−1^·s^−1^ < µ < 4400 V^2^·cm^−1^·s^−1^. The Dirac point at a bias between −40 V and −60 V indicates a strong *n*-doping that is favored by the vacuum and the electron irradiation (this measurements was performed inside a SEM, post imaging).

**Figure 4:**
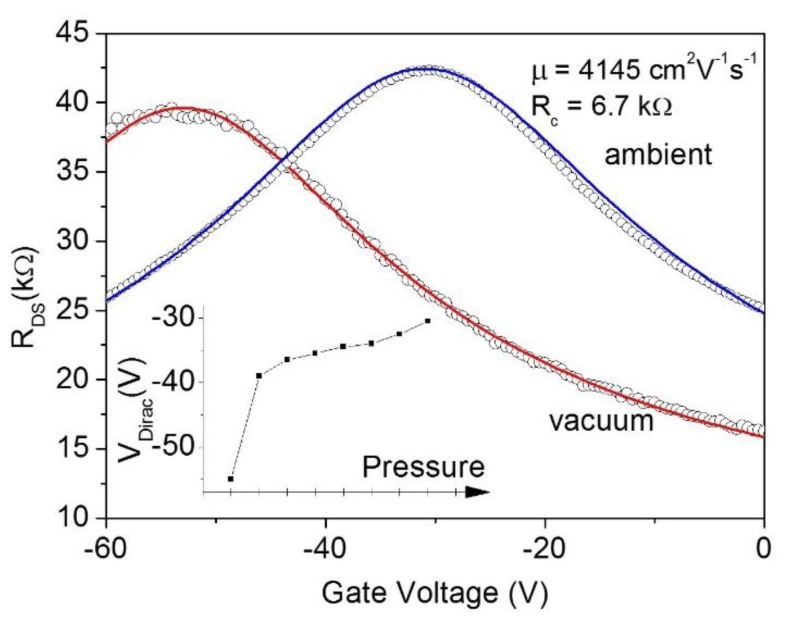
The effect of the pressure variation from high vacuum to ambient conditions on the *R*_DS_ vs. *V*_Gate_ curve reported in Figure 3a. Solid lines are the fitting curves. Inset: evolution of the Dirac point for increasing pressure.

As soon as the devices are exposed to air, the graphene collects adsorbates that, generally acting as *p*-dopants, shift the Dirac point towards positive biases, increase the contact resistance and reduce the carrier mobility [6,35,36,37,38,39]. Figure 4 compares the transfer characteristics of the device of Figure 3c measured in a high vacuum and soon after exposure to air. From the fit of the model, we extracted the contact resistance in air, as *R*_contact_ ≈ 6.7 kΩ a value 35% larger than the value in the high vacuum, while the carrier mobility was reduced to µ ≈ 4100 V^2^·cm^−1^·s^−1^. The inset shows the evolution of the Dirac point from −55 V in the high vacuum to −30 V in air. This observation confirms the importance of performing the electrical measurements in-situ when studying irradiation effects to distinguish the electron beam from other environment-induced phenomena.

### 3.2. Effect of Electron Beam Irradiation

**Figure 5:**
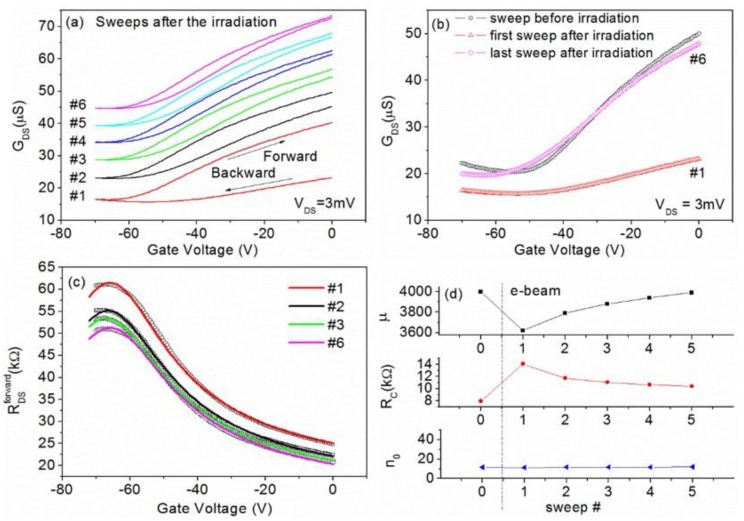
Effect of electron irradiation on *R*_DS_ vs. *V*_Gate_ of graphene field effect transistors (GFETs) characterized in Figure 3c. (**a**) Six successive sweeps recorded soon after the electron irradiation. Curves have been shifted for clarity. (**b**) Comparison of the first and sixth sweep after the 10 s e-beam exposure with that measured on unexposed device. (**c**) Forward sweep of selected measurements and relative fitting curves according to the model [32]. (**d**) Summary of parameters extracted by fitting of the curves corresponding to forward sweeps.

In the following, we consider the effect of electron beam irradiation on the GFETs. In particular, we consider electron beam energy up to 10 keV, i.e., the energy range typically used for SEM imaging. Larger energy (about 30 keV) is normally used for e-beam lithography or imaging in STEM mode. The irradiation was performed on an area of 20 µm × 20 µm, covering most of the graphene channel, with constant beam current *I*_beam_ = 0.2 nA. We used an exposure time of 10 s, which resulted in an electron irradiation dose of about 30 e^−^/nm^2^. Differently from other works [27], we performed post-irradiation electrical measurements directly in the SEM chamber, thus avoiding the aforementioned effects of air. Results obtained in six successive electrical sweeps, after a 10 s electron irradiation at 10 keV, are reported in Figure 5a. The complete (forward and backward) sweeping between 0 V and −70 V evidences an important hysteresis that decreases with successive electrical sweeps. The appearance of the hysteresis is easily explained by mobile electrons that are trapped in the gate oxide during e-beam exposure and that screen the gate voltage, while the hysteresis reduction can be caused by their withdrawal by the channel during the successive voltage sweeps [40,41]. By comparing the transfer characteristic before the electron irradiation to the first and sixth sweep measured after the 10 s exposure (Figure 5b), we observe that the device initially has a significant variation in the channel conductance, with considerably reduced gate modulation and reduced carrier mobility, while after successive sweeps it returned to its initial state apart a marginal shift of the Dirac point. To quantitatively analyze the evolution after e-beam exposure (see Figure 5c), we used the model of [32] to estimate the transport parameters, which are summarized in Figure 5d. The carrier mobility is reduced by the 10 s e-beam irradiation from 4000 V^2^·cm^−1^·s^−1^ to about 3600 V^2^·cm^−1^·s^−1^ (as obtained from the first sweep measurement). The initial value is restored by the successive sweeps. A consistent behavior is shown by the total resistance, which is increased by the irradiation and recovers with an increasing number of sweeps. The increase in total resistance, as a consequence of the e-beam irradiation, has also been observed on chemical vapor deposition (CVD) grown graphene [42]. Figure 5d reports the effect of irradiation on the contact resistance that is increased by about 70% by the exposure and is smoothly restored by successive sweeps. Noticeably, the irradiation seems to have a negligible effect on the intrinsic carrier concentration n_0_. Mobility and resistance degradation can be explained as increased long-range coulomb scattering [43] by electrons stored in the gate oxide during e-beam exposure (damaging of graphene seems to have a minor contribution); such electrons are gradually removed by voltage application during successive sweeps, and pristine conditions are partially recovered.

## 4. Conclusions

We realized graphene-based field effect transistors on a Si/SiO_2_ substrate with Nb/Au metallic bilayers as contacting electrodes. Electrical characterization evidenced high-quality devices with carrier mobility as high as 4000 cm^2^·V^−1^·s^−1^ and specific contact resistivity of about 19 kΩ·µm^2^. The effect of 10 keV electron irradiation, with a dose of 30 e^−^/nm^2^, on the transport properties has been reported, evidencing a significant reduction in carrier mobility and an increase in contact resistance. Finally, we show here that, for low energy irradiation, the pristine conditions are almost restored after several electrical sweeps, which we explain as a gradual removal of electrons piled up in the gate oxide during e-beam exposure.
